# Normal Cyclic Variation in CO_2_ Concentration in Indoor Chambers Decreases Leaf Gas Exchange and Plant Growth

**DOI:** 10.3390/plants9050663

**Published:** 2020-05-23

**Authors:** James Bunce

**Affiliations:** Adaptive Cropping Systems Laboratory, USDA-ARS, Beltsville, MD 20705-2350, USA; buncejames49@gmail.com

**Keywords:** CO_2_, light, photosynthesis, plant growth, gas exchange, stomatal opening, stomatal closing, CO_2_ variation

## Abstract

Attempts to identify crop genetic material with larger growth stimulation at projected elevated atmospheric CO_2_ concentrations are becoming more common. The probability of reductions in photosynthesis and yield caused by short-term variation in CO_2_ concentration within elevated CO_2_ treatments in the free-air CO_2_ enrichment plots raises the question of whether similar effects occur in glasshouse or indoor chamber experiments. These experiments were designed to test whether even the normal, modest, cyclic variation in CO_2_ concentration typical of indoor exposure systems have persistent impacts on photosynthesis and growth, and to explore mechanisms underlying the responses observed. Wheat, cotton, soybeans, and rice were grown from seed in indoor chambers at a mean CO_2_ concentration of 560 μmol mol^−1^, with “triangular” cyclic variation with standard deviations of either 4.5 or 18.0 μmol mol^−1^ measured with 0.1 s sampling periods with an open path analyzer. Photosynthesis, stomatal conductance, and above ground biomass at 20 to 23 days were reduced in all four species by the larger variation in CO_2_ concentration. Tests of rates of stomatal opening and closing with step changes in light and CO_2_, and tests of responses to square-wave cycling of CO_2_ were also conducted on individual leaves of these and three other species, using a leaf gas exchange system. Reduced stomatal conductance due to larger amplitude cycling of CO_2_ during growth occurred even in soybeans and rice, which had equal rates of opening and closing in response to step changes in CO_2_. The gas exchange results further indicated that reduced mean stomatal conductance was not the only cause of reduced photosynthesis in variable CO_2_ conditions.

## 1. Introduction

The concentration of CO_2_ in the atmosphere has increased from about 280 μmol mol^−1^ in 1900 to over 407 μmol mol^−1^ currently [1] and is projected to continue to increase rapidly [2]. Higher than current CO_2_ concentrations often increase photosynthesis and growth of C_3_ species, and often increase crop yields [3]. Cultivar differences in yield response to elevated CO_2_ were found in many of the major C_3_ crop species, including wheat, soybeans, rice, barley, and beans [4,5,6,7,8,9,10,11,12,13,14,15,16,17,18,19,20,21], cf. [22], and this may provide an avenue to increase future yields. Because of this possibility, the screening of cultivars for yield increases at elevated CO_2_ has become more common [5,7,8,9,10,11,16,17,18].

Field-based screening for CO_2_ responsiveness in free-air carbon dioxide enrichment (FACE) systems has the advantage of larger experimental areas than many indoor facilities, which allow more lines to be compared simultaneously, and can have otherwise natural field conditions of weather and soil. FACE systems were used in several species to screen cultivars for CO_2_ responsiveness [5,6,7,9,14,18]. However, FACE systems generally have large short-term variation in CO_2_ concentration for elevated CO_2_ treatments [23]. This recent review [23] concluded that short-term variation in CO_2_ concentrations in FACE systems reduce plant growth relative to that in more constant elevated CO_2_ environments such as open top chambers. In that review, it was proposed that reduced growth occurred because of reduced photosynthesis at least partly caused by reduced stomatal conductance. Slower plant growth caused by variable CO_2_ raises the question of whether the superior response of a genotype to elevated CO_2_ in a FACE system could reflect better tolerance to CO_2_ variation rather than a better response to elevated CO_2_. It also adds uncertainty to current estimates of the amount of increased plant growth to be expected as atmospheric CO_2_ increases.

Controlled environment facilities such as growth cabinets, glasshouses, and tunnels can provide elevated CO_2_ conditions with much smaller short-term CO_2_ variation than FACE systems. However, not all such systems have the same CO_2_ control characteristics. For example, some air-tight sunlit systems use variable flow valves such as mass flow controllers to control CO_2_, while on–off control valves are more common in glasshouses and artificially lighted chambers. In many cases, the controlling CO_2_ analyzers are outside of the plant compartment, with samples of air pumped to closed analysis cells through tubing and water traps, which result in lags in control despite rapid-response analyzers. The CO_2_ control limits are often given as mean ± x μmol mol^−1^, as detected by the remote analyzers, but “x” may be the standard deviation or the maximum deviation. It is most often not specified which type of deviation “x” indicates, and values of “x” are sometimes not even provided.

**Figure 1:**
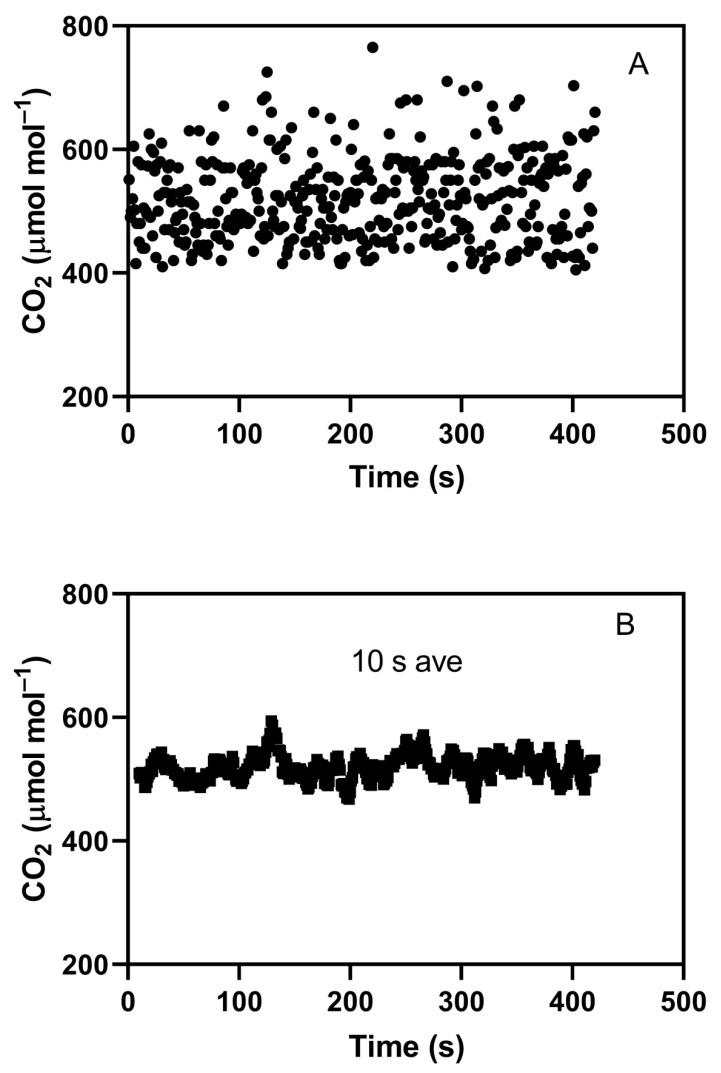
CO_2_ concentrations measured inside a controlled-environment chamber using an open path CO_2_ analyzer sampling chamber air for 0.1 s every 1 s (**A**), and concentrations calculated using a 10 s running average (**B**). The CO_2_ concentration was under the control of an external CO_2_ analyzer that sampled chamber air continuously and was used to control an on–off solenoid valve injecting pure CO_2_ into the chamber at 0.5 L per minute.

An example of an indoor chamber with on–off CO_2_ control showing a large degree of short-term CO_2_ variation is shown in Figure 1A. In this case, an M-18 chamber, interior dimensions 90 × 180 cm, with an interior height of 190 cm (EGC Inc., Chagrin Falls, OH, USA) was controlled with a TC3 controller (EGC Inc., Chagrin Falls, OH, USA). CO_2_ for control was sampled with an external WMA-4 CO_2_ analyzer (PP Systems, Amesbury, MA, USA) just outside the chamber, with analyzer output sent to the TC3 controller, which utilized proportional-integral-derivative PID control of an on–off solenoid valve. Air for CO_2_ control purposes was sampled from a shaded, ventilated box about 30 cm above the top of a full soybean canopy with a leaf area index of about 4. Pure CO_2_ was added to the chamber at the outlet of the air circulation fans mounted in the chamber side walls. Chamber air flow was downward in the plant compartment and upward through the side walls, which contained the temperature-control heat exchangers. For short-term CO_2_ analysis, air was sampled for 0.1 s at 1 s intervals using a LiCor open-path CO_2_ analyzer (LI-7500, Li-Cor, Inc., Lincoln, NE, USA) mounted horizontally 10 cm above the center of the plant canopy. The target CO_2_ concentration was 515 μmol mol^−1^. The standard deviation of CO_2_ detected by the WMA-4 analyzer was 15 μmol mol^−1^, while that detected by the Li-Cor analyzer was 68 μmol mol^−1^, with mean values of 515 and 520 μmol mol^−1^, respectively. The same 0.1 s data, but with a 10 s running average applied, is shown in Figure 1B. This running average also produced a standard deviation of about 15 μmol mol^−1^. A standard deviation of 15 μmol mol^−1^ is in the low range of those reported for elevated CO_2_ treatments in indoor chambers. This example indicates that actual short-term variation in CO_2_ in indoor chambers may routinely be much larger than detected by the analyzer controlling CO_2_ injection and documenting the CO_2_ control, because of averaging during sampling by the controlling analyzer. The primary purpose of this paper is to test whether even the normal and modest cyclic variation in CO_2_ concentration that occurs in most indoor chambers has an impact on plant growth through its effect on stomatal conductance and photosynthesis.

It is well known that stomatal closing after a decrease in light is often more rapid than opening after an increase in light [24]. If that were also true for opening and closing responses to CO_2_ changes, variation in CO_2_ could decrease mean stomatal conductance and photosynthesis, depending on the frequency of changes in CO_2_. In these experiments, two different amplitudes of cyclic variation in CO_2_ concentration, as detected by an open path analyzer, were tested for persistent differences in photosynthesis and stomatal conductance, and for aboveground biomass production in four crop species, cotton, rice, soybean, and wheat, in indoor chambers. Tests of rates of leaf stomatal opening and closing to large step changes in CO_2_ and light were made in these four species and three other species, grain amaranth, smooth pigweed, and velvet leaf. Two C_4_ species, grain amaranth and smooth pigweed, were tested because stomatal conductance response to CO_2_ is often stronger in C_4_ than in C_3_ species [24]. The step changes in CO_2_ were used to test whether stomatal conductance responses to the normal cyclic CO_2_ variation correlated with differences in rates of stomatal opening and closing in response to CO_2_. Step changes in light were used to test whether relative rates of opening and closing with changes in CO_2_ were correlated with rates of opening and closing in response to changes in light. Impacts of larger amplitude cycles of CO_2_ on stomatal conductance and photosynthesis were also assessed in order to further examine the possible role of stomatal conductance in limiting photosynthesis. These later tests utilized square-wave cycles of CO_2_ such that photosynthesis could be measured at the end of each half-cycle, which was not possible with triangular-wave cycles.

## 2. Results

### 2.1. Responses of Growth to CO_2_ Cycle Amplitude

**Figure 2:**
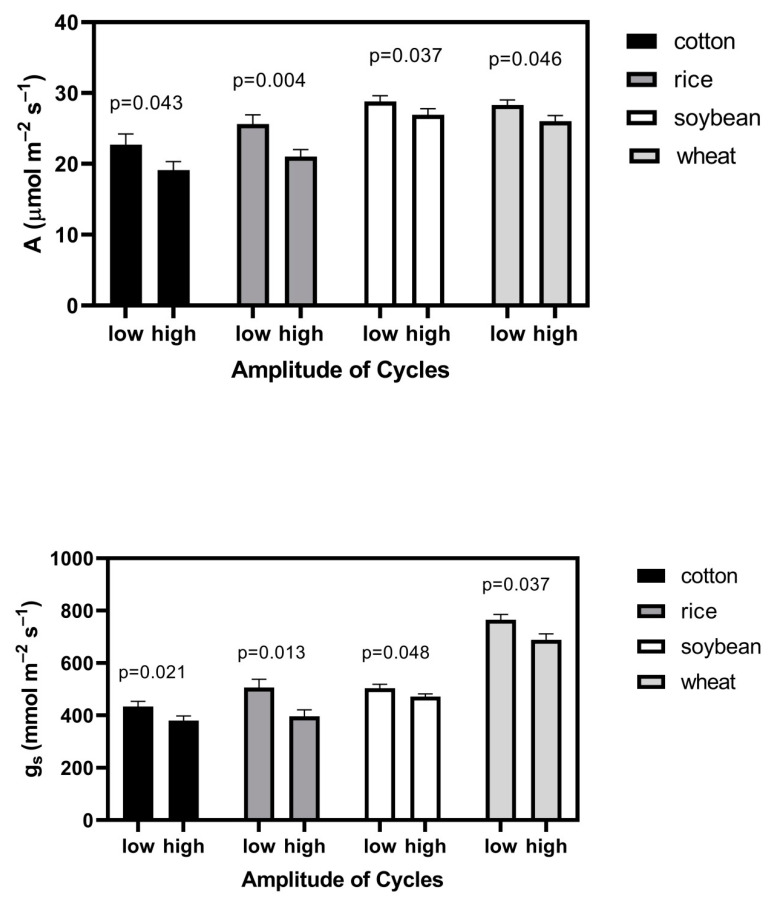
Leaf photosynthesis, A, and stomatal conductance, g_s_ measured at constant CO_2_ of 560 μmol mol^−1^, 26 °C, 1000 μmol m^−2^ s^−1^ photosynthetic photon flux density, and a water vapor pressure deficit of 1.5 kPa for four species grown with lower and higher amplitudes cycles of CO_2_ (see text for details). “*p*” indicates the probability of a greater F value, using ANOVA, with four chamber means per species per treatment, with each chamber value representing a mean value for six individual plants per species. Bars indicate SD.

The aboveground dry mass of plants was significantly less in all four species when there were larger amplitude cycles in CO_2_ (Table 1). Both leaf photosynthesis and stomatal conductance were lower when plants were grown with the larger CO_2_ fluctuation (Figure 2). The relative effect of larger CO_2_ cycles on photosynthesis was very similar to that on stomatal conductance in each species (Figure 2). Substomatal CO_2_ concentrations did not differ significantly between the CO_2_ cycle treatments in any species, with mean (SD) values for low amplitude and high amplitude cycles of 436 (8) and 440 (10) μmol mol^−1^, respectively, in cotton, 450 (13) and 441 (11) in rice, 447 (11) and 446 (14) in soybean, and 466 (8) and 469 (10) in wheat.

### 2.2. Rates of Stomatal Opening and Closing in Response to Step Changes in CO_2_ and Light

In order to test whether lower stomatal conductance in response to increased variation in CO_2_ resulted from slower stomatal opening than closing, rates of opening and closing in response to changes in CO_2_ were determined. These opening and closing rates were compared with rates of stomatal conductance responses to changes in light levels that had the equivalent effects on values of steady-state stomatal conductance.

Stomatal responses to changes in both CO_2_ and light were essentially linear with time, after initial lag periods of 2 to 7 min of no change. Lag periods for opening responses were usually longer than those for closing responses (not shown). The final transition from changing stomatal conductance to constant conductance was abrupt in all cases. The opening and closing times reported include the lag periods.

For changes in CO_2_, opening times when going from 800 to 400 μmol mol^−1^ were, with two exceptions, longer than closing times (400 to 800 μmol mol^−1^), by factors of about 1.5 to 2 (Table 2). The two exceptions were soybean and rice, where opening times did not differ from closing times. The two C_4_ species were unexceptional compared to the five C_3_ species.

For changes in light, results were even more variable among species. Closing was faster than opening in only three species, grain amaranth, rice, and velvet leaf. Opening and closing times were nearly equal to each other in three species, soybean, cotton, and smooth pigweed. In wheat, opening was faster than closing (Table 2).

### 2.3. Responses to Square-Wave Cycles of CO_2_ (400 and 800 μmol mol^−1^)

#### 2.3.1. Stomatal Conductance

**Figure 3:**
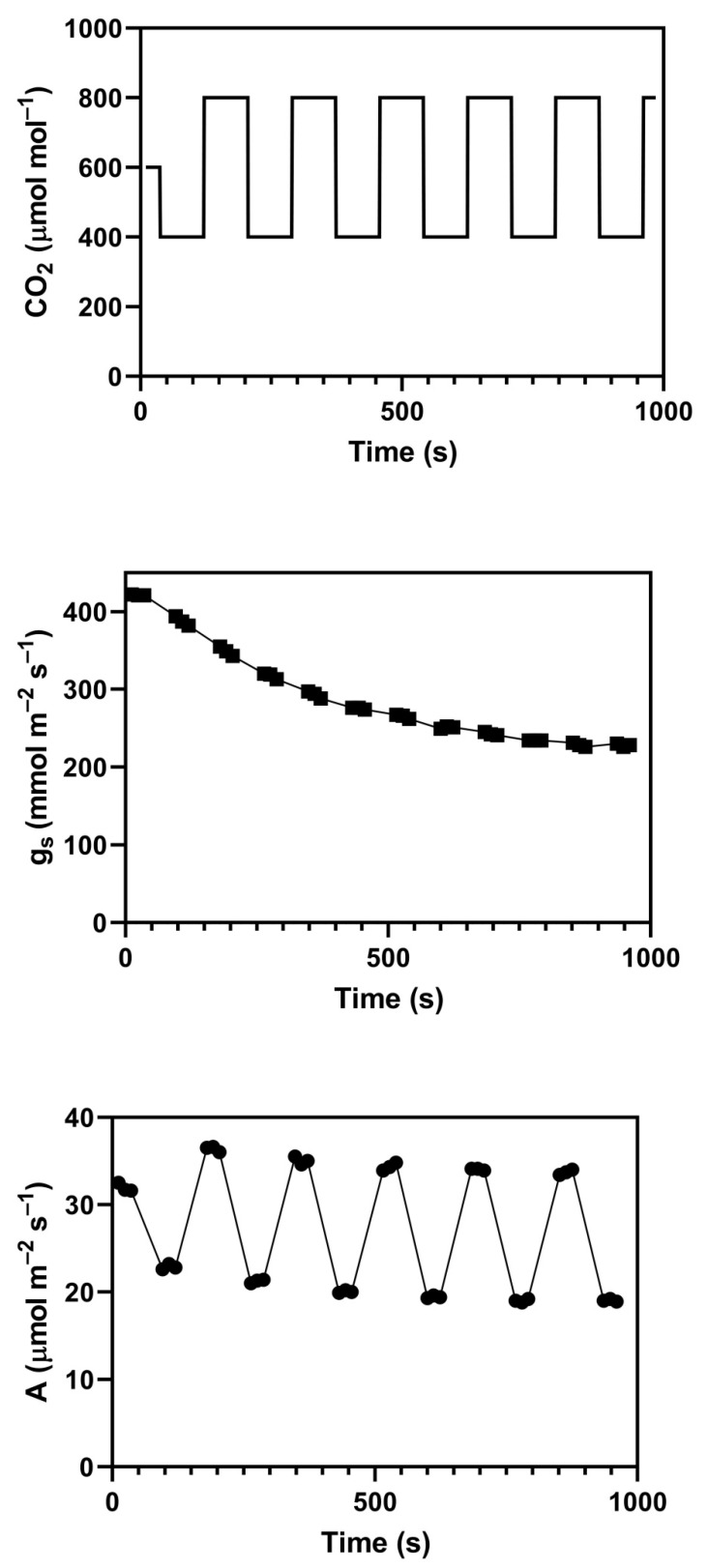
Time course of stomatal conductance, g_s_, and photosynthesis, A in a rice leaf during square-wave cycles of CO_2_ between 400 and 800 μmol mol^−1^. Three samples of g_s_ and A were taken when CO_2_ was stable at either 400 or 800 μmol mol^−1^, following initial measurements made at 600 μmol mol^−1^.

There was a clear distinction between the C_3_ and C_4_ species in responses of stomatal conductance to the square-wave cycles of CO_2_. In the two C_4_ species, grain amaranth and smooth pigweed, the final stomatal conductance equaled the mean of the steady-state conductance values at 400 and 800 μmol mol^−1^ (Table 3). This conductance value was somewhat larger than the steady-state stomatal conductance value at 600 μmol mol^−1^. In the C_3_ species, the final stomatal conductance values were in all cases equal to or less than the steady-state values at 800 μmol mol^−1^ (Table 3), and substantially less than the steady-state values at 600 μmol mol^−1^. An example time course of changes in stomatal conductance of rice during the cyclic CO_2_ treatment is given in Figure 3, and shows a gradual decrease in conductance in this case.

#### 2.3.2. Photosynthesis

**Figure 4:**
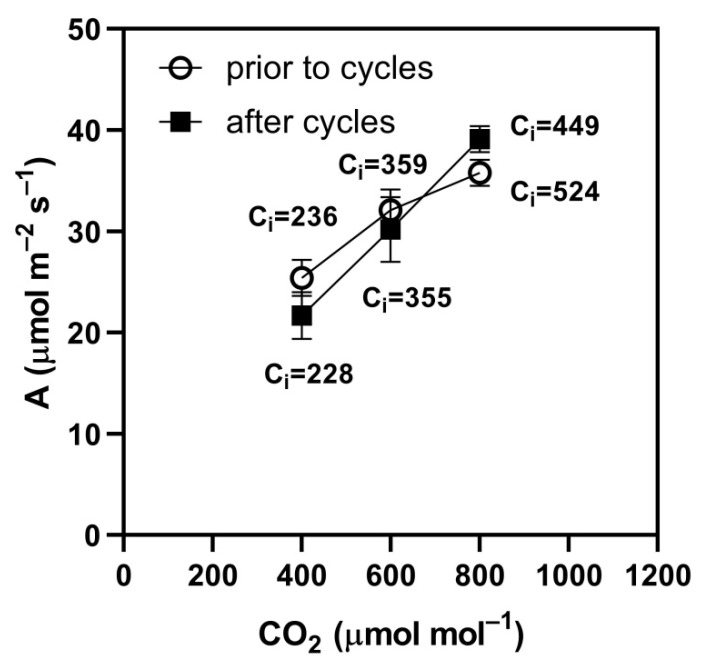
Photosynthesis, A measured at three external CO_2_ concentrations before and after 15 min of square-wave cycling of CO_2_ between 400 and 800 μmol mol^−1^. Each point is the mean of four leaves of soybean. Bars represent SD. Mean substomatal CO_2_ concentrations (C_i_) are indicated near each data point.

The final value of photosynthesis measured at constant 600 μmol mol^−1^ was less than the initial steady-state value at that concentration in three of the seven species, wheat, rice, and soybean (Table 4), but only by 6% to 8%, and was not different in the other four species. The relative reduction in photosynthesis in these three species was less than the relative reduction in stomatal conductance, which was 15% to 19%. On the other hand, the final average value of photosynthesis at 600 μmol mol^−1^ (i.e., the mean rate during the final 400 to 800 μmol mol^−1^ cycle) was in all species lower than the initial steady-state rate of photosynthesis at 600 μmol mol^−1^, by 8 to 14% (Table 4). This fairly small overall reduction resulted primarily from lower rates at 400 μmol mol^−1^ during the last cycle compared to initial steady-state rates at 400 μmol mol^−1^. In the case of soybean, higher rates occurred at 800 μmol mol^−1^ during the last cycle than the initial steady-state rates at 800 μmol mol^−1^ (Figure 4), which also illustrates that the higher rates after cycling occurred despite lower C_i_. In cotton and both C_4_ species, higher photosynthetic rates at 800 μmol mol^−1^ also occurred after the last cycle (Table 5). Only wheat had lower rates at 800 μmol mol^−1^ during the last cycle than occurred in the initial steady-state measurement (Table 5).

## 3. Discussion

**Figure 5:**
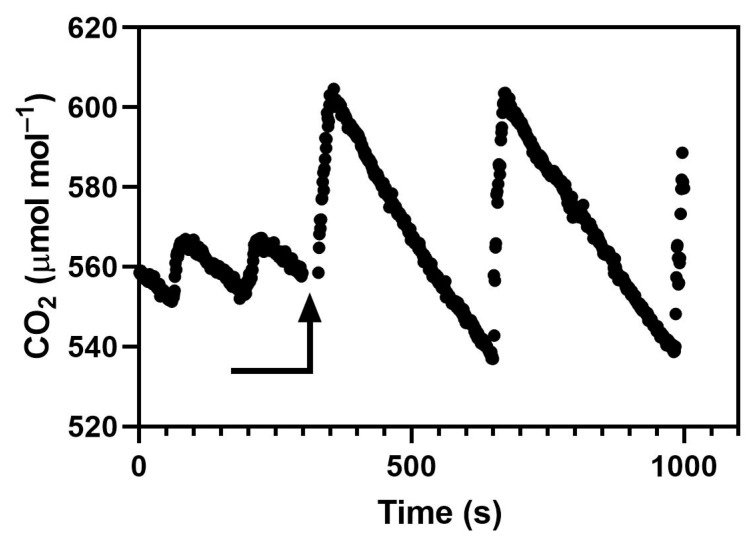
CO_2_ measured sequentially inside two controlled environment chambers with an open path CO_2_ analyzer. The arrow indicates the time when the analyzer was moved between chambers. The chambers differed in the rate of CO_2_ flow during injection, but had identical control systems.

Chambers in which CO_2_ addition is controlled by an on–off valve will have cyclic CO_2_ concentrations. The amplitude of the cycle depends on the lag in the CO_2_ measurement system, the flow rate of injected CO_2_, and the rate of loss of CO_2_ from the chamber, whether from leakage or from plant usage. The striking difference in CO_2_ variation between the examples shown in Figure 1A and Figure 5 was caused by differences in CO_2_ use rate caused by differences in canopy leaf area, not by the CO_2_ control systems. The leaf area index was about 4 in Figure 1 and less than 0.5 in Figure 5. Experiments screening lines of crops for the CO_2_ responsiveness of yield in indoor chambers would have canopy leaf areas more like Figure 1 than Figure 5 most of the time. At a minimum, variation in CO_2_ as shown in Figure 1A would reduce mean photosynthesis relative to a constant mean CO_2_ because of the curvilinear response of photosynthesis to CO_2_ [23]. Our experimental results also show that cyclic variation in CO_2_ such as would occur in all indoor chambers and glasshouses with on–off CO_2_ control decreases photosynthesis and growth compared to a more nearly constant concentration. The smaller amplitude CO_2_ cycles used in this experiment and shown in Figure 5 require very careful balancing of the CO_2_ injection rate with the plant use of CO_2_, and would not be practical to achieve in long-term studies of plant growth. Thus, short-term variation in CO_2_ sufficient to inhibit photosynthesis and growth may frequently occur in indoor chambers with CO_2_ addition, but would not be apparent with the usual CO_2_ monitoring systems. Of course, for indoor chambers even “ambient” CO_2_ treatments usually require CO_2_ addition when plants are large, so variation in CO_2_ could affect plants in both “ambient” and “elevated” treatments, but whether such effects would be equal among CO_2_ treatments is unknown. In temperature gradient chambers, the standard deviation of CO_2_ measured with a closed cell analyzer was 17–18 μmol mol^−1^ in the ambient chambers and 36–37 μmol mol^−1^ in the elevated chambers [13]. I know of no examples of measurement of short-term CO_2_ variation for air-tight sunlit chambers, but presumably “ambient” and “elevated” treatments in sunlit chambers would have identical CO_2_ control systems and probably similar CO_2_ variation. Thus, indoor chambers and sunlit chambers contrast with field-based systems like free-air carbon dioxide enrichment (FACE) systems, where “ambient” treatments would have much less short-term variation in CO_2_ than elevated treatments [23]. I do not know of information about short-term CO_2_ variation in ambient and elevated CO_2_ glasshouse compartments. The persistently reduced photosynthesis and stomatal conductance, and reduced plant growth in the four species observed in these experiments with only modest cyclic variation in CO_2_ provides a possible explanation for slower plant growth in elevated CO_2_ in FACE systems compared with open top chamber (OTC) systems, although reduced photosynthesis in FACE has yet to be demonstrated experimentally. The results presented here make it unlikely that long-term exposure to CO_2_ variation in FACE would eliminate its negative effects on leaf gas exchange. The reductions in biomass production due to cycling of CO_2_ in these experiments ranged from about 10% to 20% in cotton, rice, soybean, and wheat, which is smaller than the approximately 35% reduction summarized from FACE experiments [23]. However, the peak-to-peak variation in CO_2_ in these experiments was less than 80 μmol mol^−1^, while in FACE systems it was often more than 200 μmol mol^−1^, and the FACE experiments covered a much longer period of plant growth.

The results of responses to step changes in environment clearly indicated that lower stomatal conductance resulting from CO_2_ variation was unrelated to whether stomatal opening was slower than closing in response to step changes in environment. Lower stomatal conductance at 600 μmol mol^−1^ CO_2_ occurred after cycles of CO_2_ in all C_3_ species examined, but not in either C_4_ species. The lower stomatal conductance after repeated cycles of CO_2_ in the C_3_ species suggests that the cycling resulted in long lags in stomatal reopening. This is similar to slow stomatal reopening after treatments consisting of brief pulses of high CO_2_ in wheat and rice [25].

The reduction in the final average value of photosynthesis at 600 μmol mol^−1^ (i.e., throughout the final 400 to 800 μmol mol^−1^ cycle) compared with the initial steady-state rate of photosynthesis at 600 μmol mol^−1^, was 8% to 14% (Table 3) in all of the species in this study. “Triangular” cycles of CO_2_ applied to cotton and wheat in open top chambers in the field similarly reduced photosynthesis measured at 550 μmol mol^−1^ by 7% to 17% in cotton and wheat flag leaves (Tables 1 and 3 in [26]). Holtum and Winter [27] found larger, about 30% reductions in photosynthesis in two tree species in response to “sawtooth” (triangular) cycles of CO_2_, but provided no information about stomatal conductance.

The apparent nonstomatal inhibition of photosynthesis in the four crop species grown with the larger amplitude of cyclic CO_2_ variation could possibly be caused by patchy stomatal closure. Steady-state photosynthesis models, even when considering slower stomatal opening than closing, do not account for the observations [28]. Complete closure of stomata in patches would essentially stop both CO_2_ and H_2_O exchange from the patches and reduce photosynthesis and stomatal conductance by the proportion of leaf surface area of the closed patches, without there being any change in calculated values of substomatal CO_2_ [29,30]. Patchy stomatal behavior frequently occurs in response to sudden environmental changes [31,32], so it seems possible that sudden changes in CO_2_ concentration could cause patchy stomatal conductance. Reopening of closed patches may also have a substantial lag period, consistent with the prolonged inhibition of gas exchange seen in response to both pulses of CO_2_ [25] and observed in this study.

On the other hand, the large amplitude square-wave cycles of CO_2_ in this study probably did not induce patchy stomatal closure, as evidenced by the lack of reduction in photosynthesis in most species when measured at 800 μmol mol^−1^. Patchy stomatal closure would have resulted in equal relative reductions in photosynthesis measured at both 400 and 800 μmol mol^−1^, which did not occur in this experiment. Modeling suggested that stomatal conductance remaining at the steady-state value at the high CO_2_ concentration might explain significantly reduced photosynthesis during square-wave cycles of CO_2_ [28]. That pattern of stomatal response occurred in all of the C_3_ species in this study in response to square-wave CO_2_ cycling. However, in this study, in C_3_ species the square-wave CO_2_ cycling resulted in a shift in the response of A to C_i_, which in some cases actually increased photosynthesis at the highest external concentration despite lower C_i_ (e.g., Figure 4, Table 5). This response was not previously reported, but suggests a loosening of the limitation to photosynthesis imposed by electron transport processes after exposure to low CO_2_.

## 4. Materials and Methods

### 4.1. Response of Growth to CO_2_ Cycling

Cotton (*Gossypium hirsutum* cv. Delta Pine 555), rice (*Oryza sativa* cv. Akitakomachi), soybean (*Glycine max* cv. Clark and wheat (*Triticum aestivum* cv. Choptank) plants were grown in two M-12 growth chambers (EGC, Chagrin Falls, OH, USA) both maintained at 26/20 °C day/night air temperature, a dewpoint temperature of 18 °C, with 1000 μmol m^−2^ s^−1^ photosynthetic photon flux density (PPFD) from high pressure sodium and metal halide lamps for 12 h per day. The chamber interior dimensions were 91 × 120 cm, with an interior height of 120 cm. Chamber air was mixed at 5 m^3^ per minute. Plants were grown from seed, one per 10 cm square pot, in pots filled with 1.9 L of a medium-grade vermiculite and flushed daily with a complete nutrient solution. In each chamber run, there were six pots of each species, with pots evenly spaced across the bed. Plants were harvested at 20 days after planting in soybean, 21 days in wheat, 22 days in rice, and 23 days in cotton. The maximum leaf area index was less than 0.5. Both chambers had the same types of CO_2_ control systems, consisting of a WMA-4 CO_2_ analyzer outside the chamber, with CO_2_ addition by an on–off solenoid valve controlled by a PID controller (model CN76000, Omega Engineering, Stamford, CT), which adjusted the amount of time that the solenoid was on to achieve mean CO_2_ of 560 μmol mol^−1^. Two different standard deviations of CO_2_, 4.5 or 18.0 μmol mol^−1^, were achieved in the two chambers by having different CO_2_ injection flow rates when the solenoid valves were open, resulting in more overshooting of CO_2_ in one chamber than the other despite the PID control. The two CO_2_ injection rates were approximately 0.2 and 0.8 L per minute. An open path CO_2_ analyzer (LI-7500, LiCor, Inc., Lincoln, NE, USA) sampling CO_2_ for 0.1 s, every 1 s was used to characterize the CO_2_ cycling within both chambers several times during each approximately 3 week chamber run (Figure 5). There were four chamber runs, with the CO_2_ variation treatments switched between chambers for each run.

Rates of photosynthesis and stomatal conductance were measured 19 days after planting using a CIRAS-3 portable photosynthesis system with the leaf cuvette inside the chambers. Cuvette air temperature and leaf-to-air water vapor pressure difference were set to match the growth conditions, and the chamber light system provided the growth PPFD to the leaves inside the cuvette. The analysis CO_2_ concentration was set to 560 μmol mol^−1^. Gas exchange parameters were recorded within a few minutes of placing leaves into the cuvette, before any change in stomatal conductance caused by the switch from cyclic to constant CO_2_ occurred. Measurements were made on upper, mature leaves of all plants near midday.

### 4.2. Rates of Response to Step Changes in CO_2_

Plants of cotton, rice, soybean and wheat, as well as velvet leaf (Abutilon theophrasti), grain amaranth (*Amaranthus hypochondriacus* × *hybridus* cv. Plainsman), and smooth pigweed (*Amaranthus hybridus*) were grown under the same conditions as previously described, except at 400 ± 15 (S.D.) μmol mol^−1^ CO_2_. Plants were grown from seed, one plant per pot. Pots were filled with a medium grade vermiculite and flushed daily with a complete nutrient solution. Leaf gas exchange measurements conducted on recently fully expanded leaves. Gas exchange measurements were all made at 26 °C, with a VPD of about 1.5 kPa, using a CIRAS-3 photosynthesis system (PP Systems, Amesbury, MA, USA). Plants and the measurement cuvette were inside the growth chamber. Light for the gas exchange was provided by LED lamps set at 38% red, 37% green, and 25% blue for determination of rates of stomatal opening and closing and photosynthetic responses to programmed cycles of CO_2_. These percentages of red, green and blue give the closest approximation to sunlight for these LEDs.

For step changes in CO_2_, light was 1500 μmol m^−2^ s^−1^, and CO_2_ was stepped from 400 to 800 μmol mol^−1^ (or the reverse) until stomatal conductance responded and then became stable, and then switched to the opposite CO_2_ until stomatal conductance responded and then became stable again. For step changes in light, CO_2_ was kept at 400 μmol mol^−1^, light was initially 1500 μmol m^−2^ s^−1^, and then reduced to between 200 and 500 μmol m^−2^ s^−1^, depending on the species. The lower light level was based on initial tests of the PPFD required to reduce stomatal conductance to approximately that at high light at 800 μmol mol^−1^ CO_2_ for each species. Rates of stomatal opening and closing caused by changes in CO_2_ and light were determined using 3 to 5 leaves per species. Stomatal conductances were considered stable when changes in conductance of less than 10 mmol m^−2^ s^−1^ occurred in two minutes.

### 4.3. Gas Exchange Responses to Large Amplitude Cycles of CO_2_

Steady-state values of assimilation (A) and stomatal conductance (g_s_) were first measured at 1500 μmol m^−2^ s^−1^ PPFD, at 400, 600, and 800 μmol mol^−1^ CO_2_ at 26 °C, with a VPD of about 1.5 kPa. Leaves were then exposed to square-wave cycles of CO_2_ between 400 and 800 μmol mol^−1^, with a period of 168 s until A and g_s_ were stable. They became stable in less than 15 min of cycling of CO_2_ in all cases. The 168 s period was chosen to ensure that gas exchange rates had stabilized after each switch of CO_2_, i.e., to overcome instrumental lags, so that accurate A and g_s_ values could be recorded at the end of each half cycle. This stability is illustrated in Figure 3, where three determinations of A and g_s_ were stable after each step in the cycles. At the end of the CO_2_ cycling, photosynthesis was again measured at 600 μmol mol^−1^ for comparison with initial values at that concentration. These tests were conducted on 3 to 5 leaves per species.

## 5. Conclusions

Stomatal opening was not universally slower than closing in response to CO_2_ changes among species, nor in response to changes in PPFD. Rates of opening and closing caused by changes in PPFD were not good predictors of rates of opening and closing caused by changes in CO_2_. Stomatal conductance under cyclic CO_2_ treatments was not well predicted by rates of opening and closing in response to step changes in CO_2_. Even modest short-term variation in CO_2_, in this case cycles with a standard deviation of 18 μmol mol^−1^, with a mean value of 560 μmol mol^−1^, caused a persistent apparent nonstomatal inhibition of photosynthesis, in addition to lower stomatal conductance, and resulted in slower plant growth in all four species that were tested.

## Figures and Tables

**Table 1:** Total aboveground dry mass in four species grown with low or high amplitudes of CO_2_ cycling (see text for details). Harvests were at 20 days after planting in soybean, 21 days in wheat, 22 days in rice, and 23 days in cotton. There were four replicates for each species, with each replicate consisting of the mean value for six plants per treatment. Standard deviations are in parenthesis.

Species	Mass, Low Amplitude	Mass, High Amplitude	Probability of >F
	(g)	(g)	
Cotton	1.95 (0.26)	1.57 (0.10)	0.028
Rice	0.563 (0.043)	0.436 (0.083)	0.035
Soybean	3.61 (0.18)	3.28 (0.17)	0.032
Wheat	0.552 (0.022)	0.485 (0.017)	0.009

**Table 2:** Times required to open and close stomata with change in CO_2_ between 400 and 800 μmol mol^−1^ at a PPFD of 1500 μmol m^−2^ s^−1^, and to open or close between PPFDs of 1500 and 200–500 μmol m^−2^ s^−1^, depending upon species, at a CO_2_ concentration of 400 μmol mol^−1^. Within species, means followed by different letters are significantly different at *p* = 0.05 using ANOVA. Standard deviations are in parenthesis.

Species	Time (Minutes) to Open or Close			
	Change in CO_2_		Change in PPFD	
	Open	Close	Open	Close
Soybean	21 a (1.7)	22 a (1.6)	11 b (0.6)	11 b (0.6)
Cotton	18 a (2.5)	12 b (0.7)	13 b (2.1)	13 b (2.0)
Rice	12 a (2.4)	12 a (2.3)	11 a (0.5)	6 b (0.6)
Wheat	18 a (1.5)	11 b (1.0)	7 c (1.5)	15 a (2.1)
Velvet leaf	27 a (2.0)	18 b (1.0)	16 b (1.0)	11 c (1.5)
Grain amaranth	22 a (2.6)	12 b (1.5)	8 b (1.5)	4 c (1.0)
Smooth pigweed	15 a (0.8)	10 b (2.0)	5 c (1.2)	4 c (1.2)

**Table 3:** Steady-state stomatal conductance (mmol m^−2^ s^−1^) at three CO_2_ concentrations at PPFD = 1500 μmol m^−2^ s^−1^, and final stomatal conductance after square-wave cycles of CO_2_ between 400 and 800 μmol mol^−1^. Within species, means followed by different letters are significantly different at *p* = 0.05 using repeated measures ANOVA. Standard deviations are in parenthesis.

Species	Steady-State Stomatal Conductance			Final Cycle
CO_2_ μmol mol^−1^	400	600	800	mean = 600
Soybean	380 (25) a	318 (12) b	297 (14) c	258 (18) d
Cotton	265 (19) a	235 (15) b	224 (11) c	213 (10) d
Rice	425 (28) a	300 (26) b	250 (19) c	247 (19) c
Wheat	500 (45) a	365 (23) b	323 (24) c	312 (19) c
Velvet leaf	683 (38) a	516 (18) b	478 (17) c	478 (19) c
Grain amaranth	283 (22) a	211 (16) b	185 (15) c	230 (20) b
Smooth pigweed	251 (18) a	155 (18) b	120 (16) c	187 (17) b

**Table 4:** Photosynthetic rates (μmol m^−2^ s^−1^) measured at 600 μmol mol^−1^ CO_2_ and 1500 μmol m^−2^ s^−1^ PPFD before and after square-wave cycles of CO_2_ between 400 and 800 μmol mol^−1^, and during the last cycle. Within species, means followed by different letters are significantly different at *p* = 0.05 using repeated measures ANOVA. Standard deviations are in parenthesis.

Species	Before	After	During
Soybean	31.1 (1.5) a	29.3 (0.7) b	28.3 (0.6) c
Cotton	35.0 (1.5) a	35.0 (1.4) a	32.1 (1.3) b
Rice	30.0 (1.3) a	28.3 (1.7) b	25.7 (1.6) c
Wheat	38.3 (1.4) a	35.3 (1.9) b	33.0 (1.3) c
Velvet leaf	38.3 (1.8) a	38.0 (1.9) a	34.2 (1.9) b
Grain amaranth	33.7 (1.8) a	33.1 (1.9) a	31.1 (1.7) b
Smooth pigweed	39.1 (2.0) a	39.3 (1.8) a	36.2 (1.8) b

**Table 5:** Photosynthetic rates (μmol m^−2^ s^−1^) and substomatal CO_2_ concentrations (μmol mol^−1^) measured at 800 μmol mol^−1^ CO_2_ and 1500 μmol m^−2^ s^−1^ PPFD before and after square-wave cycles of CO_2_ between 400 and 800 μmol mol^−1^. Within species, means for each parameter followed by different letters are significantly different at *p* = 0.05 using repeated measures ANOVA. Standard deviations are in parenthesis.

Species	Before		After	
	A	C_i_	A	C_i_
Cotton	38.0 (2.2) b	427 (31) a	41.1 (1.8) a	412 (22) a
Rice	35.3 (1.6) a	526 (17) a	34.5 (2.1) a	529 (34) a
Wheat	44.1 (3.7) a	545 (25) a	41.0 (4.0) b	520 (20) a
Velvet leaf	40.4 (2.1) a	587 (24) a	41.1 (2.6) a	576 (36) a
Grain amaranth	33.1 (2.0) b	411 (15) a	36.3 (2.5) a	412 (13) a
Smooth pigweed	38.3 (2.1) b	362 (15) a	40.7 (2.7) a	360 (25) a
